# Association between age and outcomes of evidence-based feeding protocol versus usual care in critically ill patients: secondary analysis of a multicenter cluster-randomised controlled trial

**DOI:** 10.1186/s40560-026-00942-y

**Published:** 2026-09-21

**Authors:** Qiuhui Wang, Jin Ke, Yizhe Chen, Hongyang Xu, Dingye Wu, Lu Ke, Fengming Liang, Yang Chen

**Affiliations:** 1 Department of Critical Care Medicine, The Affiliated Wuxi People’s Hospital of Nanjing Medical University, Wuxi Medical Center, Nanjing Medical University, No. 299 Qingyang Road, Wuxi, 214023 China; 2 Department of Critical Care Medicine, Jinling Hospital, Affiliated Hospital of Medical School, Nanjing University, No. 305 East Zhongshan Road, Nanjing, 210002 China; 3 Liverpool Centre for Cardiovascular Science at University of Liverpool, Liverpool John Moores University and Liverpool Heart and Chest Hospital, William Henry Duncan Building, 6 West Derby Street, Liverpool, L7 8TX UK; 4 Department of Cardiovascular and Metabolic Medicine, Institute of Life Course and Medical Sciences, University of Liverpool, William Henry Duncan Building, 6 West Derby Street, Liverpool, L7 8TX UK

**Keywords:** Enteral nutrition, Nutritional therapy, Age, Mortality, Critical illness

## Abstract

**Background:**

Nutrition therapy is essential in critically ill patients, yet large randomised trials have shown inconsistent results regarding the effect of an evidence-based feeding protocol. The NEED trial showed a feeding protocol could effectively promote delivery of enteral nutrition (EN) but did not reduce mortality. Age-related physiological differences may modify responses to nutritional therapy. This post hoc analysis of the NEED trial evaluated whether age modifies the association between implementation of an evidence-based feeding protocol and clinical outcomes among critically ill patients.

**Methods:**

This secondary analysis used data from the multicentre cluster-randomised NEED trial conducted across intensive care units (ICUs) in China. Adult patients newly admitted to participating ICUs were assigned to active implementation of a feeding protocol or usual care. Age was analyzed as both a continuous variable and predefined categories (≤65, 66–75, and ≥76 years). The primary effect-modification analysis evaluated the treatment–age interaction for 28-day all-cause mortality. Secondary outcome analyses, including organ failure (OF), persistent multiple OF, ICU length of stay, ICU-free days, infection within 7 days, and feeding intolerance, were exploratory. Mixed-effects Cox and logistic regression models were used to assess treatment effects and age-treatment interactions.

**Results:**

Among 2672 patients included in the analysis (median age 62 years [IQR 49–74]; 1798 [67.3%] male), the effect of evidence-based feeding protocol on 28-day mortality varied with age. Treatment benefit attenuated progressively with increasing age, with each 10-year increase associated with a higher adjusted hazard ratio (HR) for mortality (1.28; 95% CI 1.13–1.46; continuous interaction *P* < 0.001). In age-stratified analyses, the intervention reduced mortality in patients aged ≤65 years (adjusted HR 0.64; 95% CI 0.44–0.92) but not in those aged 66–75 years (adjusted HR 1.01; 95% CI 0.64–1.60) or ≥76 years (adjusted HR 1.29; 95% CI 0.89–1.88; categorical interaction *P* = 0.010). An exploratory analysis showed a similar age-dependent pattern for persistent multiple OF (interaction *P* = 0.017). Results were consistent in per-protocol and sensitivity analyses.

**Conclusions:**

In this secondary analysis, associations between an evidence-based feeding protocol and clinical outcomes differed across age groups. These exploratory and hypothesis-generating findings suggest potential age-related heterogeneity in treatment response, which requires confirmation in future prospective studies.

*Research registration unique identifying number (UIN) of the original NEED trial*: ISRCTN Registry, Registry number: ISRCTN 12233792, registered on November 24, 2017.

**Supplementary Information:**

The online version contains supplementary material available at https://doi.org/10.1186/s40560-026-00942-y.

## Introduction

Nutritional therapy is a core component of supportive care in critically ill patients, yet the optimal strategy remains uncertain. Both underdelivery and overdelivery of energy and protein may be harmful [1], and feeding intolerance commonly limits the ability to achieve prescribed targets [2]. Contemporary guidelines therefore advocate a progressive, individualised approach to early enteral nutrition (EN) and cautious use of parenteral nutrition, with gradual advancement toward energy goals over the first week [3].

However, large randomised trials evaluating alternative nutrition strategies in critically ill patients have produced inconsistent effects on clinical outcomes [4–6]. Clinical heterogeneity within intensive care unit (ICU) populations may partly explain this variability [7], as treatment effects may not be uniform across patients.

The NEED trial, a multicentre cluster-randomised study, evaluated active implementation of an evidence-based feeding protocol versus usual practice in newly admitted ICU patients [8]. Although the intervention improved delivery processes, it did not reduce mortality overall, raising the possibility of variation in patient responses within the ICU population.

Age is a plausible source of heterogeneity in critical illness. Older patients more often have reduced physiological reserve, sarcopenia, multimorbidity, and impaired gastrointestinal tolerance [9], which may influence how patients respond to nutritional therapy [10]. Whether associations between evidence-based feeding protocols and clinical outcomes differ across age groups remains unclear. We therefore performed a post hoc analysis of the NEED trial to examine age-related differences in the association between feeding protocol implementation and clinical outcomes.

## Methods

### Ethical review

The NEED trial was approved by the Ethics Committee of Jinling Hospital (approval number 22017NZKY-019-02) and was registered prior to enrolment at the ISRCTN registry (ISRCTN12233792). Randomisation was performed at the ICU level before individual patient enrolment. Eligible patients admitted to participating ICUs were subsequently approached for enrolment, and written informed consent was obtained from all participants or their legally authorised representatives before enrolment. No patients were enrolled under a waiver of informed consent. The current secondary analysis used deidentified data in accordance with the original ethical approval and complied with the principles of the Declaration of Helsinki. The reporting of this study followed the Consolidated Standards of Reporting Trials (CONSORT) guidelines, as appropriate [11].

### Study design and clinical setting

This study represents a secondary analysis of data derived from the NEED trial, a multicentre, cluster-randomised controlled study conducted in China. A total of 97 ICUs were randomised in the original trial, of which 7 withdrew before enrolling any patients, leaving 90 ICUs that participated in patient enrolment. Of these, 88 ICUs (43 intervention and 45 control) contributed patients to the present analysis. Randomisation was performed at the ICU level using computer-generated random numbers, with the allocation sequence generated by an independent statistician. Participating ICUs were stratified according to province and ICU type (emergency, medical, surgical, neurosurgical, or general ICU). ICUs were allocated in a 1:1 ratio to either the evidence-based feeding protocol implementation group or the control group. Allocation concealment was ensured until hospital participation was confirmed. No important changes to the trial outcomes or analyses were made after trial commencement, and no interim analyses or stopping guidelines were specified for this trial. Details of the original trial design, protocol, and statistical analysis plan have been reported previously [8].

The original trial enrolled 2772 adult patients newly admitted to participating ICUs between March 2018 and July 2019. Eligible patients were aged ≥18 years and enrolled within 24 h of ICU admission, with one or more organ failures (Sequential Organ Failure Assessment [SOFA] score ≥2 in any organ system), an expected ICU stay >7 days, and a low likelihood of resuming oral intake within 3 days. In the NEED trial, ICUs were randomised to either active implementation of a structured feeding protocol or continuation of usual local nutritional practice. The intervention protocol provided recommendations on initiation of enteral and parenteral nutrition, assessment of feeding tolerance, and adjustment of nutritional delivery to achieve predefined energy (25–30 kcal/kg/day) and protein (1.2–2.0 g/kg/day) targets. Detailed descriptions of the intervention protocol are provided in the Supplementary Methods and Supplementary Figure S1. The analytic cohort was identical to that of the primary NEED trial publication, with complete baseline age data available for all included participants. Patients and members of the public were not involved in the design, conduct, reporting, or dissemination plans of this secondary analysis. Detailed eligibility criteria in original trial are provided in the Supplementary Methods.

### Study population and study outcomes

**Fig. 1:**
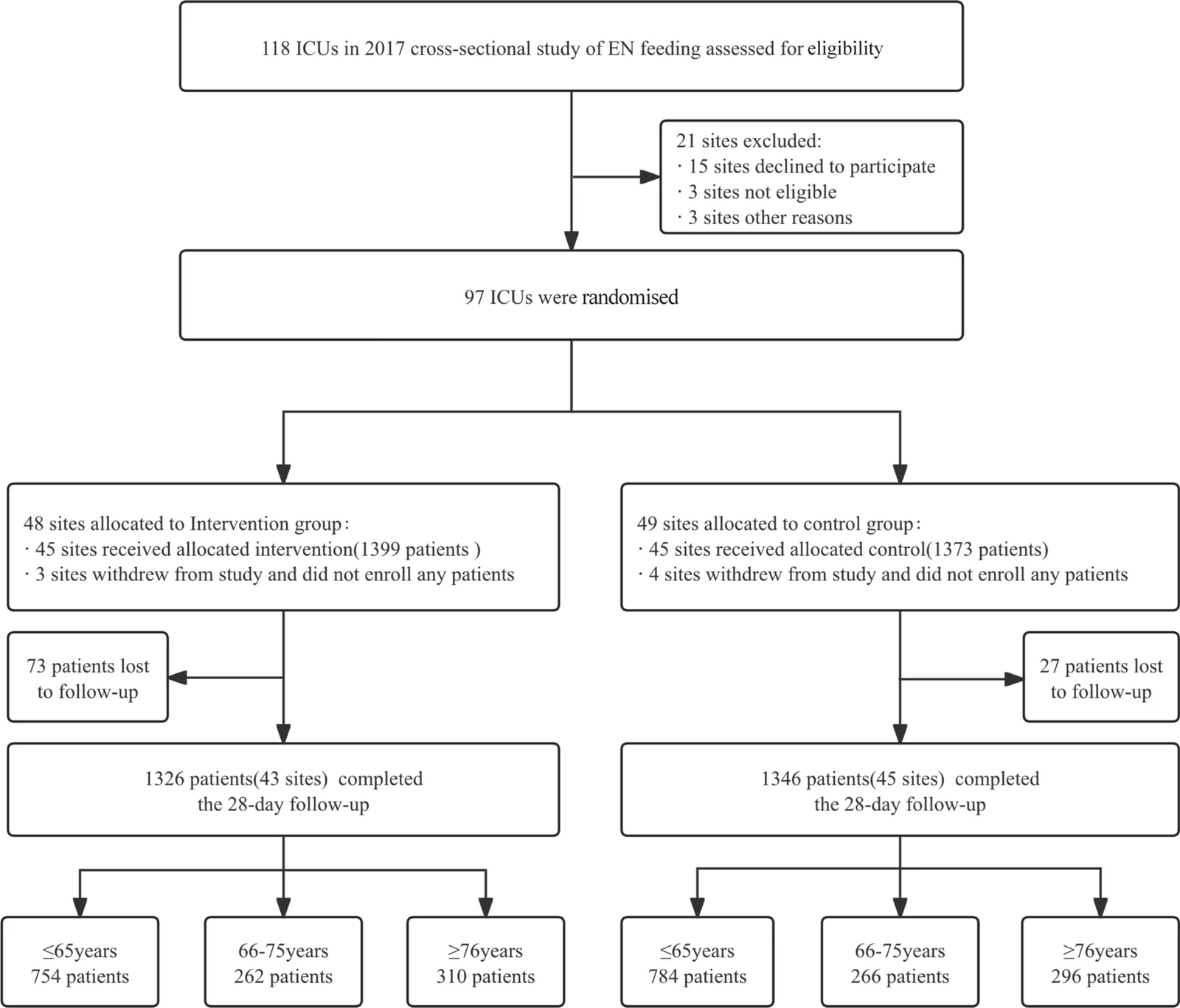
CONSORT flow diagram. EN, enteral nutrition; ICU, intensive care unit

Among the 2772 patients enrolled in the original NEED trial, 100 patients (73/1399 [5.2%] in the intervention group and 27/1373 [2.0%] in the control group) were lost to follow-up before day 28 and therefore had incomplete ascertainment of 28-day vital status. Across age strata, 57/1595 (3.6%) patients aged ≤65 years, 24/552 (4.3%) aged 66–75 years, and 19/625 (3.0%) aged ≥76 years were lost to follow-up. A total of 2672 patients were therefore included in the present analysis. The detailed participant flow is shown in Fig. 1.

The primary outcome was 28-day all-cause mortality after ICU admission, as centrally adjudicated in the original NEED trial. Secondary outcomes included ICU length of stay, 28-day ICU-free days, persistent organ failure (OF), new-onset OF, persistent multiple OF, new-onset multiple OF, and new-onset infection within 7 days after ICU admission. Persistent OF was defined as a SOFA score ≥2 in a given organ system on day 7 [12]. As the neurological component of SOFA may be unreliable in sedated patients, a modified five-component SOFA excluding the neurological domain was used. New-onset OF was defined as an organ system with a SOFA score <2 on day 1 that increased to ≥2 by day 7. Persistent multiple OF was defined as failure in two or more organ systems on day 7, and new-onset multiple OF as newly developed failure in two or more organ systems by day 7 among patients without multiple OF at baseline. New-onset infection within 7 days was defined as a new infection site identified on day 7 that was absent at baseline, based on available clinical and microbiological evidence. Infection status was determined at the participating sites according to the original NEED trial definitions, without separate central adjudication. For nutritional delivery measures, adequate EN target attainment on day 7 was defined as achievement of ≥60% of the prescribed energy or protein target, consistent with the predefined criterion in the original NEED trial protocol [8].

### Data collection

Baseline demographic and clinical characteristics were collected at enrolment and are summarised in Table 1. Nutrition therapy related variables, including measures of delivery, timing, and target attainment, are detailed in Supplementary Table S1.

**Table 1:** Baseline characteristics by age group and intervention

Characteristics	Age: ≤65 years (*N* = 1538)			Age: 66–75 years (*N* = 528)			Age: ≥76 years (*N* = 606)		
	Intervention (*N* = 754)	Control (*N* = 784)	SMD	Intervention (*N* = 262)	Control (*N* = 266)	SMD	Intervention (*N* = 310)	Control (*N* = 296)	SMD
Age (years)	51.0 (41.0, 59.0)	50.0 (40.0, 58.0)	0.038	70.0 (68.0, 73.0)	70.0 (68.0, 73.0)	0.038	83.0 (79.0, 86.0)	82.0 (79.0, 86.0)	0.014
Male, *n* (%)	515 (68.3)	541 (69.0)	0.015	165 (63.0)	178 (66.9)	0.083	212 (68.4)	187 (63.2)	0.110
BMI (kg/m^2^)	22.9 (21.3, 24.7)	23.1 (21.4, 25.0)	0.030	22.5 (20.8, 24.2)	22.9 (20.8, 24.8)	0.152	22.0 (20.1, 23.9)	22.3 (20.5, 24.4)	0.136
SOFA score	7.0 (5.0, 10.0)	7.0 (5.0, 10.0)	0.123	7.0 (5.0, 10.0)	8.0 (5.0, 10.0)	0.090	7.0 (5.0, 9.0)	8.0 (6.0, 10.0)	0.137
APACHE II score	16.0 (12.0, 20.0)	17.0 (12.0, 21.0)	0.055	21.0 (16.0, 25.0)	20.0 (14.0, 26.0)	0.013	20.0 (16.0, 24.0)	21.0 (17.0, 26.0)	0.113
mNUTRIC score	3.0 (2.0, 5.0)	4.0 (2.0, 5.0)	0.040	5.0 (4.0, 6.0)	5.0 (3.0, 6.0)	0.025	6.0 (5.0, 7.0)	6.0 (4.0, 7.0)	0.078
AGI grade, *n* (%)			0.285			0.293			0.272
AGI I	556 (73.7)	499 (63.6)		205 (78.2)	183 (68.8)		248 (80.0)	213 (72.0)	
AGI II	136 (18.0)	183 (23.3)		47 (17.9)	56 (21.1)		51 (16.5)	54 (18.2)	
AGI III	36 (4.8)	85 (10.8)		6 (2.3)	21 (7.9)		7 (2.3)	21 (7.1)	
AGI IV	26 (3.4)	17 (2.2)		4 (1.5)	6 (2.3)		4 (1.3)	8 (2.7)	
Primary admission diagnosis, *n* (%)			0.279			0.195			0.191
Respiratory system	211 (28.0)	188 (24.0)		81 (30.9)	94 (35.3)		120 (38.7)	130 (43.9)	
Cardiovascular system	137 (18.2)	231 (29.5)		61 (23.3)	73 (27.4)		87 (28.1)	90 (30.4)	
Neurological system	267 (35.4)	221 (28.2)		93 (35.5)	71 (26.7)		72 (23.2)	47 (15.9)	
Others	139 (18.4)	144 (18.4)		27 (10.3)	28 (10.5)		31 (10.0)	29 (9.8)	
Source of ICU admission, *n* (%)			0.081			0.168			0.175
Emergency department	302 (40.1)	312 (39.8)		100 (38.2)	99 (37.2)		160 (51.6)	127 (42.9)	
Surgical department	211 (28.0)	209 (26.7)		77 (29.4)	69 (25.9)		53 (17.1)	60 (20.3)	
Medical department	99 (13.1)	92 (11.7)		58 (22.1)	56 (21.1)		74 (23.9)	83 (28.0)	
Others	142 (18.8)	171 (21.8)		27 (10.3)	42 (15.8)		23 (7.4)	26 (8.8)	
Comorbidities, *n* (%)									
Diabetes mellitus	80 (10.6)	97 (12.4)	0.055	57 (21.8)	67 (25.2)	0.081	92 (29.7)	97 (32.8)	0.067
Hypertension	270 (35.8)	225 (28.7)	0.153	134 (51.1)	152 (57.1)	0.121	199 (64.2)	192 (64.9)	0.014
Coronary artery disease	40 (5.3)	64 (8.2)	0.114	56 (21.4)	65 (24.4)	0.073	112 (36.1)	117 (39.5)	0.070
Chronic respiratory disease	40 (5.3)	33 (4.2)	0.052	40 (15.3)	36 (13.5)	0.049	62 (20.0)	51 (17.2)	0.071
Stroke	57 (7.6)	51 (6.5)	0.041	45 (17.2)	48 (18.0)	0.023	102 (32.9)	72 (24.3)	0.191
Gastrointestinal disease	35 (4.6)	58 (7.4)	0.116	22 (8.4)	30 (11.3)	0.097	16 (5.2)	35 (11.8)	0.241
Malignant disease	13 (1.7)	20 (2.6)	0.057	17 (6.5)	19 (7.1)	0.026	11 (3.5)	19 (6.4)	0.132
Organ support therapy, *n* (%)									
Continuous renal replacement therapy	82 (10.9)	136 (17.3)	0.187	21 (8.0)	31 (11.7)	0.122	21 (6.8)	37 (12.5)	0.195
Mechanical ventilation	530 (70.3)	561 (71.6)	0.028	188 (71.8)	187 (70.3)	0.032	189 (61.0)	211 (71.3)	0.219
Vasopressors	210 (27.9)	303 (38.6)	0.231	78 (29.8)	104 (39.1)	0.197	99 (31.9)	114 (38.5)	0.138

Values are presented as median (interquartile range) for continuous variables and number (percentage) for categorical variables

*AGI* acute gastrointestinal injury, *APACHE II* Acute Physiology and Chronic Health Evaluation II, *BMI* body mass index, *ICU* intensive care unit, *mNUTRIC* modified Nutrition Risk in the Critically Ill, *SMD* standardised mean difference, *SOFA* Sequential Organ Failure Assessment

### Statistical analysis

The present secondary analysis was exploratory and was not prespecified in the protocol or statistical analysis plan of the parent NEED trial. The analysis plan for the present study, including the age strata, interaction analyses, RCS modelling, per-protocol definition, and sensitivity analyses, was developed after completion and publication of the primary NEED trial and was not separately registered or deposited in a public repository.

Patients were categorised into age groups of ≤65, 66–75, and ≥76 years, informed by clinically relevant thresholds around 65 and 75 years commonly used to characterise older and more advanced older populations in geriatric and critical care nutrition literature [10, 13]. In primary analyses, age was modelled as a continuous variable in prognostic models.

Missing data are summarised in Supplementary Table S2. Age, sex, BMI, and SOFA score were complete in the analytic cohort and therefore required no imputation. Missing baseline data were limited to APACHE II score, mNUTRIC score, and acute gastrointestinal injury (AGI) grade. Missing values for these variables were addressed using multiple imputation by chained equations with 20 imputed datasets, which were used for analyses incorporating variables with missing data. Predictive mean matching was used for continuous variables, and logistic or multinomial logistic regression was used for binary and categorical variables, respectively. The imputation model included treatment allocation and all baseline covariates included in the regression models. Baseline SOFA score was included in the imputation model when missing. SOFA-derived secondary outcomes were calculated separately from observed day 7 organ function assessments and were not included as predictors in the imputation model. The primary outcome was fully observed and was therefore not imputed. Estimates from the imputed datasets were combined using Rubin’s rules. Descriptive statistics are presented as median with interquartile range (IQR) for continuous variables and as number with percentage for categorical variables. Treatment comparisons followed the intention-to-treat (ITT) principle based on treatment assignment at randomisation.

Distributions of study outcomes, treatment delivery, nutritional target attainment, and 7-day feeding intolerance symptoms were compared between treatment groups using methods appropriate to the underlying data type. For the primary outcome, event rates were additionally calculated on a weekly basis, and rate differences (RDs) between treatment groups were estimated with corresponding 95% confidence intervals (CIs).

Potential non linear associations between age and treatment effects for the primary outcome were assessed using restricted cubic spline (RCS) analysis in multivariable mixed-effects Cox proportional hazards models adjusting for sex, body mass index (BMI), and SOFA score. Given the cluster-randomised design of the NEED trial, ICU was incorporated as a random effect to account for within-ICU correlation. Knot selection was guided by model fit criteria, including the Akaike information criterion and Bayesian information criterion. Linear specifications were adopted when non linearity was not supported. Using the same mixed-effects Cox models, treatment–age interaction terms were estimated, with effect modification quantified as the relative change in hazard ratio (HR) per 10-year increase in age.

Kaplan–Meier survival curves were constructed by treatment group within each age stratum for the primary outcome, with differences assessed using the log rank test. Crude and multivariable mixed-effects Cox proportional hazards models were used to estimate HRs and 95% CIs within defined age groups, with adjustment for age, sex, BMI, and SOFA score. ICU was incorporated as a random effect in all Cox models to account for within-ICU correlation. Treatment–age group interactions were included to formally assess effect modification. The proportional hazards assumption was assessed using Schoenfeld residuals and was not violated (Supplementary Table S3).

Secondary binary outcomes were analysed using mixed-effects logistic regression models to estimate odds ratios (ORs) and 95% CIs, with ICU incorporated as a random effect to account for within-ICU correlation. Multivariable models were adjusted using age, sex, BMI, and SOFA score. Effect modification by age was assessed by including treatment–age group interaction terms. Continuous secondary outcomes were assessed for normality and compared between treatment groups within each age group using appropriate tests.

A per-protocol analysis was conducted for the primary outcome, excluding patients who died or were discharged from the ICU within 7 days and thus could not complete the planned 7-day nutrition therapy. Primary analyses were repeated, modelling age as a continuous variable and estimating treatment effects within defined age groups using mixed-effects Cox proportional hazards models with ICU included as a random effect. An additional analysis using inverse-probability-of-censoring weighting (IPCW) was performed to account for potential selection bias related to exclusion of patients who died or were discharged within 7 days. Stabilised IPCW was estimated using baseline characteristics and applied in mixed-effects Cox proportional hazards models with ICU included as a random effect.

Sensitivity analyses were performed using alternative multivariable models that incorporated ICU as a random effect. For sensitivity models not incorporating APACHE II score, the primary adjustment set of age, sex, BMI, and SOFA score was retained. Given the residual baseline imbalance in primary admission diagnosis and AGI grade, a sensitivity model further adjusted the primary model for these two variables. Given that both SOFA and Acute Physiology and Chronic Health Evaluation II (APACHE II) scores reflect illness severity, sensitivity models incorporating APACHE II and modified Nutrition Risk in the Critically Ill (mNUTRIC) scores were specified without simultaneous adjustment for SOFA score. The mNUTRIC score was calculated without interleukin-6 using baseline variables collected at enrolment, including age, APACHE II score, SOFA score, number of comorbidities, and time from hospital admission to ICU admission. Additional models accounted for treatment-related variables, including continuous renal replacement therapy, mechanical ventilation, and vasopressor use, as well as their combination. To assess the potential impact of differential loss to follow-up, extreme-case sensitivity analyses were additionally performed for 28-day mortality among patients aged ≤65 years, in whom the intervention was associated with lower mortality in the primary analysis. Mortality was recalculated under four assumptions: all patients lost to follow-up survived; all patients lost to follow-up died; a best-case scenario for the intervention, in which lost patients in the intervention group survived and those in the control group died; and a worst-case scenario for the intervention, in which lost patients in the intervention group died and those in the control group survived. Finally, to account for the stratification factors used in the original ICU-level randomisation, additional sensitivity analyses incorporated province and ICU type, first as fixed effects with ICU retained as a random effect and, in an alternative specification, as random effects together with ICU.

All statistical analyses were conducted using R version 4.2.1 (R Foundation for Statistical Computing, Vienna, Austria). A two-sided *P* value <0.05 was considered statistically significant. The sample size was determined in the original NEED trial, and no additional sample size calculation was performed for this secondary analysis.

## Results

### Baseline characteristics

A total of 2672 patients were included in the analysis (median age 62 years [IQR 49–74]; 1798 [67.3%] male). Age was well balanced between intervention and control groups within each defined age stratum. Baseline characteristics were broadly similar between treatment assignments, although residual imbalances were observed for several characteristics within individual age strata (Table 1), as may occur with ICU-level cluster randomisation. These included primary admission diagnosis, AGI grade, gastrointestinal comorbidity, and organ support therapies. Among patients aged ≥76 years, mechanical ventilation (61.0% vs. 71.3%), continuous renal replacement therapy (6.8% vs. 12.5%), and AGI grade III (2.3% vs. 7.1%) were less frequent in the intervention group despite the higher observed mortality in this group. Among patients aged ≤65 years, however, the intervention group also had lower use of vasopressors (27.9% vs. 38.6%) and continuous renal replacement therapy (10.9% vs. 17.3%). Across age strata, increasing age was associated with a greater burden of comorbidities and higher baseline severity scores. In contrast, younger patients had lower illness severity and lower rates of cardiovascular comorbidities. ICU admission source and primary diagnosis varied by age, with patients aged ≥76 years more frequently admitted from emergency departments. The use of organ support therapies was common across all age groups, although their distribution differed modestly by age. Baseline characteristics of patients included in and excluded from the analysis are presented in Supplementary Table S4, with some differences in clinical characteristics.

### Nutrition therapy delivery and target attainment

Across age groups, the intervention modified patterns of nutrition delivery. Early EN initiation within 48 h was more frequent in the intervention group, particularly among patients aged ≤65 and ≥76 years. Mean daily energy delivery over the first 7 days was similar between treatment groups within each age category. However, the intervention improved EN target attainment. In patients aged ≤65 years, the intervention group had significantly higher day 7 EN energy and protein target ratios and a greater proportion achieving ≥60% of predefined targets. In those aged 66–75 years, target attainment was numerically higher but not statistically significant. Among patients aged ≥76 years, the intervention was associated with higher day 7 EN energy target ratios and greater achievement of ≥60% of the target, whereas differences in protein targets were not significant.

### Feeding intolerance symptoms

Gastrointestinal intolerance and feeding interruption during the first 7 days are shown in Supplementary Table S5, these comparisons were exploratory and unadjusted, with no correction for multiplicity. Abdominal distension or pain occurred less frequently in the intervention group across age strata, whereas nausea or vomiting was uncommon and similar between groups. Diarrhoea rates were comparable in patients aged ≤65 years but were numerically higher in the intervention group among older patients, particularly in those aged ≥76 years (62.6% vs. 50.3%), with more frequent consecutive episodes (26.5% vs. 20.3%). Feeding interruption was similar between groups in younger patients but was more frequent in the intervention group among patients aged ≥76 years (13.9% vs. 6.8%), including consecutive interruptions (4.8% vs. 1.4%) (Supplementary Table S5).

### Nutrition related adverse events

Adverse events potentially related to nutrition therapy were uncommon, with all reported events occurring at rates below 1.0% across age groups and treatment assignments (Supplementary Table S6).

### Intention-to-treat analysis for the primary outcome

**Fig. 2:**
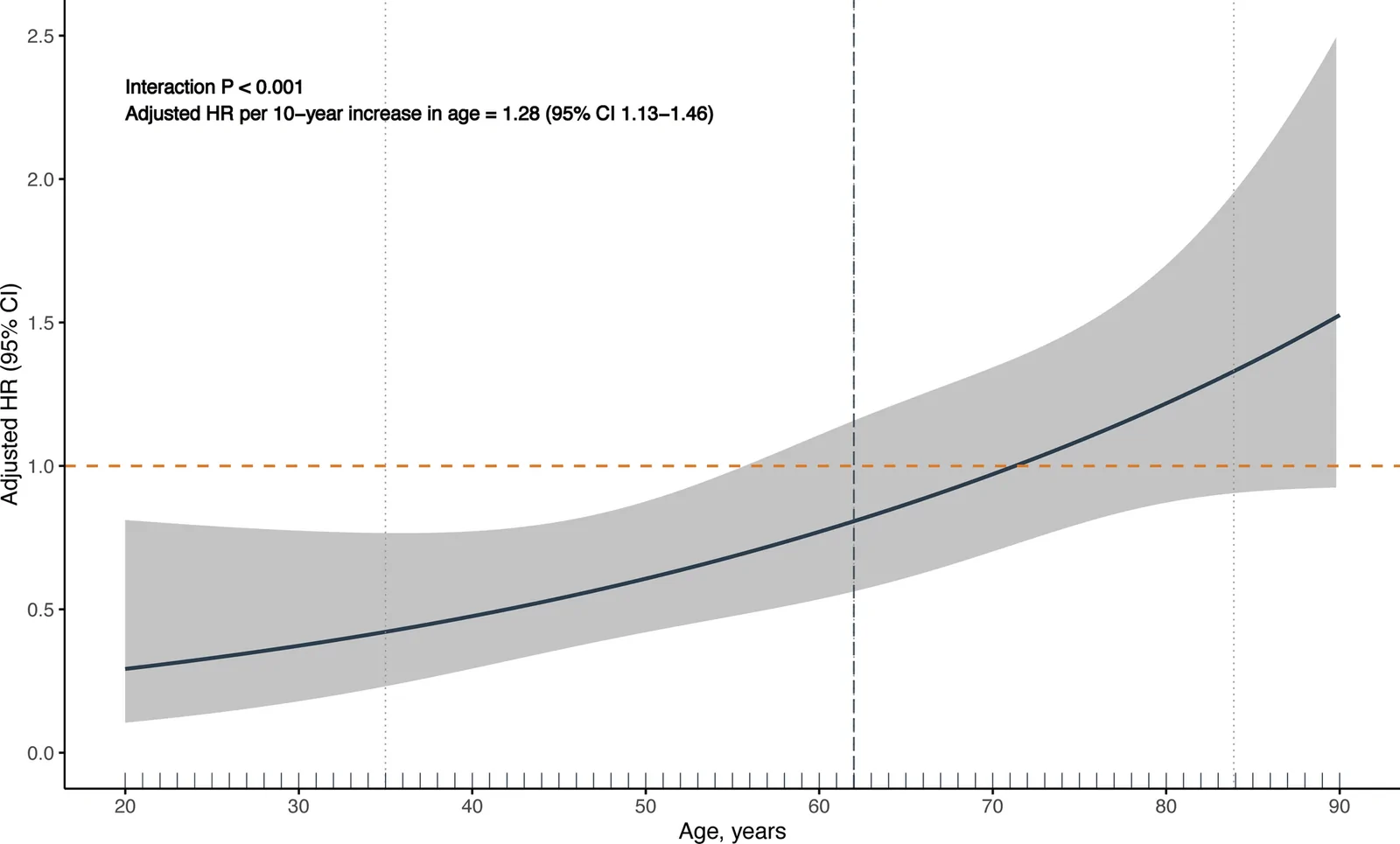
Treatment effect on the primary outcome as a function of age as a continuous variable in the intention-to-treat analysis. The solid line represents the adjusted HR for the intervention versus control at each age estimated using the three-knot restricted cubic spline model, and the shaded area represents the 95% CI. Vertical dotted lines indicate the spline knots placed at the 10th, 50th, and 90th percentiles of the age distribution. The median age of the study population was 62 years and coincided with the middle (50th-percentile) knot. The horizontal dashed line indicates the null value (HR = 1.0), and the rug plot along the *x*-axis shows the underlying age distribution. Models were adjusted for sex, body mass index, and Sequential Organ Failure Assessment score, with ICU included as a random effect to account for within-ICU clustering. The per-10-year HR and interaction *P* value shown in the figure were derived from the corresponding linear treatment-by-age interaction model after no evidence of non-linearity was identified. CI, confidence interval; HR, hazard ratio; ICU, intensive care unit

**Fig. 3:**
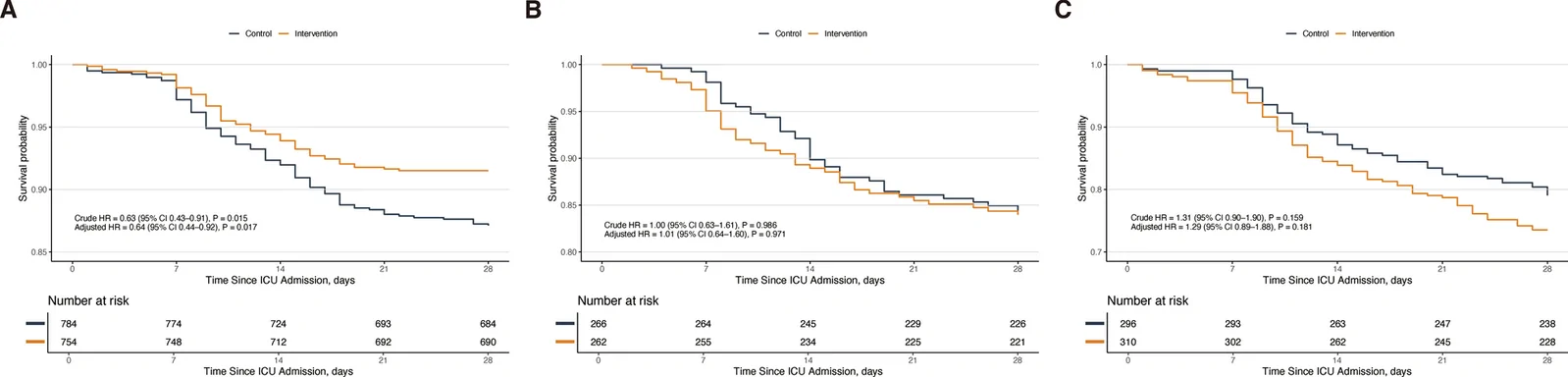
Kaplan–Meier curves for primary outcome across age groups by treatment group. This figure shows Kaplan–Meier survival curves for the primary outcome (28-day all-cause mortality after ICU admission) comparing the intervention and control groups stratified by age group over a fixed 28-day follow-up period. Panel **A** shows participants aged ≤65 years, panel **B** those aged 66–75 years, and panel **C** those aged ≥76 years. Numbers at risk are shown below each panel. Crude HRs and adjusted HRs with 95% CIs are shown on the plot. Adjusted HRs were derived from mixed-effects Cox proportional hazards models adjusted for age, sex, body mass index, and Sequential Organ Failure Assessment score, with ICU included as a random effect to account for within-ICU clustering. Log-rank *P* values are provided for comparison of survival curves. CI, confidence interval; HR, hazard ratio; ICU, intensive care unit

**Fig. 4:**
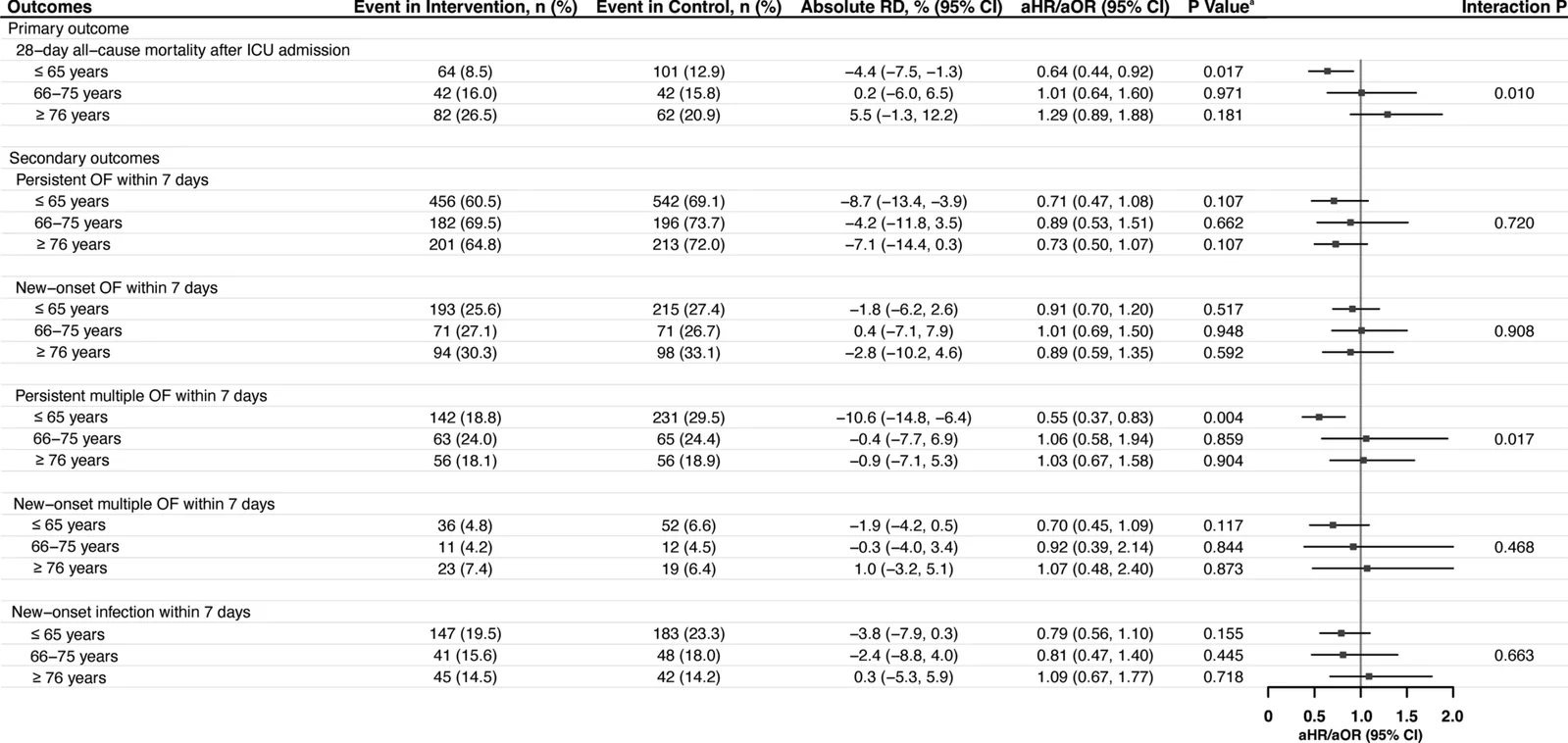
Treatment effects on the primary and binary secondary outcomes across age groups. Treatment effects are presented as adjusted HRs for 28-day all-cause mortality and adjusted ORs for binary secondary outcomes, with corresponding 95% CIs. Models were adjusted for age, sex, body mass index, and Sequential Organ Failure Assessment score, with ICU included as a random effect to account for within-ICU clustering. Absolute RDs are presented as the observed event proportion in the intervention group minus that in the control group, with corresponding 95% CIs. Negative RDs indicate a lower absolute risk with the intervention. *P* values for interaction assess treatment-effect heterogeneity across age groups. aHR, adjusted hazard ratio; aOR, adjusted odds ratio; CI, confidence interval; ICU, intensive care unit; OF, organ failure; RD, risk differences

RCS analysis with three knots (Supplementary Table S7) showed no evidence of non-linearity between age and treatment effect for the primary outcome (non-linear *P* = 0.984). Age was therefore modelled linearly, and the treatment effect varied continuously with age (Fig. 2). Kaplan–Meier curves for primary outcome are shown in Fig. 3. aHRs were lowest at younger ages and increased progressively across the age spectrum. Each 10-year increase in age was associated with a 28% increase in the adjusted HR (1.28; 95% CI 1.13–1.46; interaction *P* < 0.001). Corresponding 28-day mortality rates (Fig. 4) were 8.5% versus 12.9% in patients aged ≤65 years, 16.0% versus 15.8% in those aged 66–75 years, and 26.5% versus 20.9% in patients aged ≥76 years (intervention versus control). Weekly event rates were 2.2% versus 3.5% in the ≤65-year group (intervention versus control; RD −1.23%; 95% CI −2.10 to −0.36), 4.4% versus 4.3% in the 66–75-year group, and 7.7% versus 5.8% in the ≥76-year group (Supplementary Table S8). Consistently, in multivariable mixed-effects Cox proportional hazards models stratified by age group, the intervention reduced 28-day mortality in patients aged ≤65 years (aHR 0.64; 95% CI 0.44–0.92), but not in older age groups (66–75 years: 1.01 [0.64–1.60]; ≥76 years: 1.29 [0.89–1.88]; interaction *P* = 0.010; Fig. 4).

### Intention-to-treat analysis for the secondary outcome

All secondary outcome analyses were exploratory, and interaction *P* values were not adjusted for multiplicity. Event rates for secondary outcomes by treatment group are shown in Supplementary Table S9. Among patients aged ≤65 years, point estimates across binary secondary outcomes generally favoured the intervention. For persistent multiple OF within 7 days (Fig. 4), the intervention was associated with lower odds in patients aged ≤65 years (aOR 0.55; 95% CI 0.37–0.83), whereas the corresponding estimates were 1.06 (95% CI 0.58–1.94) in those aged 66–75 years and 1.03 (95% CI 0.67–1.58) in those aged ≥76 years (*P* for interaction = 0.017).

### Per-protocol analysis for the primary outcome

In the per-protocol analysis (*n* = 2531), after accounting for ICU-level clustering, the continuous age–treatment interaction remained significant, with each 10-year increase in age associated with a higher adjusted HR for mortality (1.24; 95% CI 1.08–1.41; interaction *P* = 0.002) (Supplementary Figure S2). Age-stratified analyses showed a more favourable treatment estimate in patients aged ≤65 years but not in older strata (interaction *P* = 0.041; Supplementary Table S10). In an additional analysis using IPCW to account for potential selection related to exclusion of early deaths or ICU discharges, the age–treatment interaction remained consistent, with similar estimates for both continuous age and age-stratified analyses (Supplementary Table S11).

### Sensitivity analyses for the primary outcome

Sensitivity analyses incorporating extended multivariable adjustment (Supplementary Table S12) showed consistent findings, with comparable effect estimates and age–treatment interaction results. Of the 100 patients lost to follow-up, 57 were aged ≤65 years (41 intervention and 16 control), 24 were aged 66–75 years (17 intervention and 7 control), and 19 were aged ≥76 years (15 intervention and 4 control). Among patients aged ≤65 years, extreme-case analyses showed that the mortality difference remained in favour of the intervention when all patients lost to follow-up were assumed to have survived (8.1% vs. 12.6%) or all were assumed to have died (13.2% vs. 14.6%). Under treatment-specific best- and worst-case scenarios, mortality was 8.1% versus 14.6% and 13.2% versus 12.6%, respectively, with the direction of the difference slightly reversed in the worst-case scenario (Supplementary Table S13). Additional sensitivity analyses accounting for the original randomisation stratification factors, including province and ICU type, yielded findings consistent with the primary analysis under both fixed- and random-effects specifications (Supplementary Table S14).

## Discussion

In this post hoc analysis of the NEED trial, associations between an evidence-based feeding protocol and clinical outcomes differed across age groups in critically ill patients. Although the intervention improved EN target attainment across age groups, more favourable treatment effects on survival were observed at younger ages, with attenuation of the estimated effect with advancing age. When age was modelled continuously, a similar age-related pattern was observed across the age spectrum. These findings were consistent across ITT and per-protocol analyses and remained robust in multiple sensitivity analyses. Exploratory analysis of persistent multiple OF demonstrated a comparable age-dependent pattern.

The original NEED trial showed that active implementation of an evidence-based feeding protocol improved several process measures of nutritional therapy, including earlier initiation of enteral nutrition and higher energy delivery, but did not reduce mortality overall [8]. Our findings suggest that the neutral overall effect observed in the original NEED trial may partly reflect heterogeneity in treatment response across age groups. Importantly, the intervention improved EN target attainment across age groups, including among patients aged ≥76 years, indicating that attenuation of clinical benefit in older patients was not simply explained by failure to deliver nutritional therapy. Instead, improved nutritional delivery may not translate into equivalent physiological or clinical benefits in patients with different levels of biological reserve. Ageing is associated with sarcopenia, reduced physiological reserve, and impaired metabolic adaptability during critical illness [14, 15]. These factors, including potential anabolic resistance and impaired responsiveness to nutritional substrates, may help explain the observed age-related differences in treatment effects; however, they were not directly measured in the NEED trial and should therefore be considered potential explanations rather than demonstrated mechanisms. Younger patients may derive greater physiological benefit from improved nutrient delivery because their anabolic response to nutritional substrates remains relatively preserved [16]. Increased energy and protein provision may have the potential to contribute to muscle protein synthesis, metabolic recovery, and organ function in patients with greater physiological reserve, although these processes were not directly assessed in the present study [17]. In contrast, older patients frequently exhibit anabolic resistance, characterised by a reduced muscle protein synthetic response to dietary protein or amino acid, which may limit the physiological benefits of increased nutrient delivery [18].

Exploratory analysis of persistent multiple OF suggested a pattern consistent with the primary outcome. Organ dysfunction represents a key intermediate outcome in critical illness and is associated with mortality [19]. Adequate nutritional treatment has been hypothesised to influence organ function through multiple pathways, including preservation of gut barrier integrity, metabolic regulation, and modulation of systemic inflammation [2]. However, these mechanisms were not directly evaluated in the present study. The observed reduction in persistent OF among younger patients may partly contribute to the more favourable treatment effect on survival. Conversely, the potential organ-protective effects of improved nutritional delivery may be attenuated with advancing age. Elderly patients often present with a greater burden of comorbidities and frailty [20], which may impair their ability to recover from OF despite improved nutritional support. However, this secondary finding was not adjusted for multiplicity and should be considered hypothesis generating.

In the present study, exploratory analyses of feeding intolerance showed a lower incidence of abdominal distension or pain in the intervention group. These comparisons were unadjusted and were not corrected for multiplicity and should therefore be interpreted cautiously. Early EN has been proposed to support gastrointestinal function through effects on gut mucosal integrity and motility; however, these biological effects were not directly measured in the current dataset [21]. We also observed a higher incidence of diarrhoea among patients aged ≥76 years in the intervention group, together with increased feeding interruptions. Although the present study was not designed to determine whether increased nutritional exposure contributed to these gastrointestinal events, this finding raises the possibility that improved delivery may have different consequences in older patients with reduced gastrointestinal tolerance. This pattern may reflect that increased enteral nutrition delivery raises intestinal osmotic load in vulnerable patients [22]. Age-related alterations in gastrointestinal physiology, including impaired motility, altered microbiota, and reduced absorptive capacity, have been proposed as potential contributors to feeding intolerance in older patients [23]. Increased nutritional exposure may therefore lead to more frequent gastrointestinal symptoms and interruptions of enteral feeding, which may reduce effective nutrient delivery and attenuate the potential benefits of nutritional therapy on organ recovery [24]. However, given the exploratory nature and multiple unadjusted comparisons, these potential mechanisms cannot be inferred from the present findings and require further investigation.

The evidence base for nutritional therapy in critical illness has also evolved since the NEED trial was conducted in 2018–2019. Subsequent trials, including EFFORT Protein and NUTRIREA-3 [25, 26], have raised uncertainty regarding the benefits of higher early protein and energy delivery and reinforced a more cautious, progressive approach during the acute phase of critical illness. The NEED intervention should therefore be interpreted within the nutritional practice and evidence context in which the trial was conducted, although its comparison of protocolised versus usual nutritional care remains relevant to understanding heterogeneity in response to nutritional interventions.

These findings have important clinical implications. Contemporary guidelines advocate a progressive and individualised approach to nutritional therapy in critically ill patients rather than rigid attainment of predefined caloric targets [27, 28]. Our findings extend this concept by suggesting that improvements in EN delivery may not translate into uniform clinical benefit across age groups. This may be particularly relevant in routine ICU practice, where patient characteristics such as age, frailty, physiological reserve, and tolerance to feeding vary substantially.

Rather than supporting immediate changes to nutritional practice, these findings highlight the need for further investigation of patient heterogeneity in response to nutritional interventions. From a research perspective, they further underscore the need to account for patient heterogeneity in the design and evaluation of nutritional strategies, including approaches that better capture variability in treatment response across critically ill populations.

Several limitations need consideration. First, this was a post hoc subgroup analysis of a cluster-randomised trial, and age-related effect modification was not the primary objective of the parent study. Subgroup analyses inherently carry reduced precision and an increased risk of chance findings, particularly in older strata with fewer events, and the interaction analyses were not adjusted for multiplicity. Therefore, the observed age–treatment interactions, including the apparent benefit observed among younger patients, should be interpreted cautiously and regarded as hypothesis generating rather than confirmatory, as chance findings cannot be excluded given the post hoc nature of the analysis and the lack of adjustment for multiplicity. Residual baseline imbalances within age strata should also be acknowledged, particularly for severity-related characteristics, and may have influenced the magnitude of the age-specific treatment estimates despite consistent findings in additional adjusted analyses. In addition, chronological age may not fully capture biological ageing or physiological vulnerability. Frailty, functional status, sarcopenia, and markers of anabolic resistance were not systematically assessed and could not be incorporated into the analyses; therefore, age may partly serve as a proxy for these unmeasured characteristics. Second, the intervention was implemented at the ICU level and comprised multiple components. We were unable to disentangle the relative contribution of specific elements or assess implementation fidelity in detail. Third, the per-protocol analysis may introduce post-randomisation selection bias, as exclusion of patients who died or were discharged within 7 days conditions the analysis on post-randomisation events, including early mortality. Although an IPCW-adjusted sensitivity analysis showed consistent findings, residual bias due to unmeasured factors affecting early death or discharge cannot be excluded. Accordingly, per-protocol findings should be considered supportive rather than confirmatory. Differential loss to follow-up between treatment groups may also have introduced attrition bias; although the mortality difference among patients aged ≤65 years remained directionally consistent under the all-survived and all-died assumptions, it was slightly reversed under a treatment-specific worst-case scenario. Fourth, nutrition delivery and feeding intolerance variables were derived from recorded daily data and may not fully capture cumulative deficits or dynamic changes over time. In addition, gastrointestinal surgical status, which may influence feeding tolerance and enteral nutrition delivery, was not available and could not be accounted for in the analyses. Secondary outcomes were exploratory and were not adjusted for multiplicity; thus, these findings should also be considered hypothesis generating. Finally, all participating ICUs were located in China, which may limit generalisability to other healthcare settings because of differences in patient case-mix and nutritional practices. Detailed information on protein sources, including animal- versus plant-derived protein, was not collected and could not be evaluated. External validation in other healthcare settings is warranted.

## Conclusions

In this secondary analysis of the NEED trial, associations between an evidence-based feeding protocol and clinical outcomes differed across age groups. Although enteral nutrition target attainment improved, these improvements did not translate into uniform clinical outcomes. These exploratory and hypothesis-generating findings suggest potential age-related heterogeneity in treatment response, which requires confirmation in future prospective studies before informing age-specific nutritional strategies.

## Supplementary Information

Additional file 1

## Data Availability

The data supporting the findings of this study and the analysis code used for the present secondary analysis are available from the corresponding author, Yang Chen (yang.chen2@liverpool.ac.uk), upon reasonable request, subject to applicable data-sharing and institutional requirements.
