# Supplementary material for: Association between age and outcomes of evidence-based feeding protocol versus usual care in critically ill patients: secondary analysis of a multicenter cluster-randomised controlled trial
Source: J Intensive Care. 2026 Sep 21;14:103. doi: 10.1186/s40560-026-00942-y (PMC13591688; doi:10.1186/s40560-026-00942-y)
Supplement: Supplementary file 1 [file 40560_2026_942_MOESM1_ESM.docx]

**Supplementary Methods**

*Implementation of the Feeding Protocol*

An evidence-based feeding protocol was developed based on international nutrition guidelines and update literature review. The protocol was implemented in intervention ICUs through a structured implementation package.

Each participating center in the intervention group established a multidisciplinary “intervention team”, consisting of physicians, nurses, and dietitians, responsible for local implementation of the feeding protocol. Standardized educational materials, including graphical feeding algorithms and bedside checklists, were provided to facilitate protocol adherence.

Training sessions and online educational meetings were organized for investigators before and during the study period to ensure consistent protocol implementation. Graphic feeding protocols were placed at bedside locations as visual reminders for clinicians.

ICUs assigned to the control group continued routine nutritional management and were not informed of the protocol content during the study period.

# *Table of Feeding Intolerance Score*

| Points | 0 | 1 | 2 | 5 |
| --- | --- | --- | --- | --- |
| Abdominal distention/pain | None | Mild distension and no abdominal pain | Moderate distension **OR** IAP 15~20mmHg **OR** transient abdominal pain | Severe distension **OR** IAP>20mmHg **OR** persistent abdominal pain |
| Nausea/vomiting | None | Nausea but no vomiting | Nausea and vomiting without a requirement for decompression **OR** 250ml≤ GRV<500ml | Vomiting requiring gastric decompression **OR** GRV≥500ml |
| Diarrhea | None | Loose stools ≥3 times/day with 250≤ volume <500ml | Loose stools ≥3 times/day with 500≤ volume <1500ml | Loose stools ≥3 times/day with volume ≥1500ml |

Total score = Abdominal distension/pain + Nausea/vomiting + Diarrhea

0-2 points: continue enteral nutrition, increase or maintain initial speed, symptomatic treatment;

3-4 points: continue enteral nutrition, slow down the speed, reevaluate EN tolerance after 2h;

≥ 5 points: suspend enteral nutrition, reevaluate or replace the infusion route;

*Feeding Protocol*

The feeding protocol emphasized early EN and standardized escalation of nutritional therapy. Patients were evaluated for gastrointestinal function within 24 h after haemodynamic stabilization. According to gastrointestinal status and aspiration risk, nasogastric feeding or post-plyoric feeding was recommended.

EN was initiated at a low rate and progressively increased according to feeding tolerance assessment. The protocol recommended gradually increasing EN delivery toward target nutritional goals (approximately 25-30 kcal/kg/day and protein 1.2-2.0g/kg/day) when tolerated.

If EN remained insufficient and patients failed to reach approximately 60% of their nutritional targets within the first week, supplemental nutrition was recommended.

*Assessment and Management of Feeding Intolerance*

Feeding intolerance was reassessed every 4-6 h using a standardized feeding intolerance score incorporating gastrointestinal symptoms and intra-abdominal pressure. Clinical manifestations included abdominal distension, vomiting, diarrhea, abdominal pain, and other gastrointestinal complications.

Based on the feeding intolerance score, enteral nutrition was adjusted according to the severity of gastrointestinal intolerance: the feeding rate was maintained in patients with mild intolerance, reduced in those with moderate intolerance, and temporarily suspended in cases of severe intolerance.

Further diagnostic evaluation was recommended when persistent abdominal pain, vomiting, or diarrhea occurred. Differential diagnoses included disease-related gastrointestinal dysfunction, medication-related adverse effects, or infectious causes such as Clostridioides difficile infection.

Supportive measures such as prokinetic agents, post-pyloric feeding, or temporary reduction of feeding rate were recommended according to clinical conditions.

*Eligibility criteria*

Patients were eligible for inclusion when all of the following criteria were fulfilled: aged ≥18 years, ICU admission within the previous 24 hours, presence of at least one organ system failure (defined as a Sequential Organ Failure Assessment [SOFA] score ≥2 in any organ system), an anticipated ICU stay exceeding 7 days, and an expected inability to resume oral intake within 3 days.

Exact composition of the SOFA score used for OF definitions (modified five-component)

| Domain | Definition of organ failure (SOFA ≥2) | 0 | 1 | 2 | 3 | 4 |
| --- | --- | --- | --- | --- | --- | --- |
| Respiratory | PaO_2_/FiO_2_ (mmHg) | >400 | ≤400 | ≤300 | ≤200^*^ | ≤100^*^ |
| Coagulation | Platelet count ×10^9^/L | >150 | ≤150 | ≤100 | ≤50 | ≤20 |
| Hepatic | Bilirubin (mg/dL) | <1.2 | 1.2-1.9 | 2.0-5.9 | 6.0-11.9 | >12 |
| Cardiovascular | Hypotension | No hypotension | Mean arterial pressure <70 mmHg | Dopamine ≤5 or dobutamine (any dose) | Dopamine >5, epinephrine ≤0.1, or norepinephrine ≤0.1 | Dopamine >15, epinephrine >0.1, or norepinephrine >0.1 |
| Renal | Creatinine (mg/dL) | <1.2 | 1.2-1.9 | 2.0-3.4 | 3.5-4.9 | >5 |

^*^For the respiratory domain, PaO_2_/FiO_2_ thresholds of ≤200 and ≤100 apply in the presence of respiratory support.

Organ failure was defined as a score ≥2 in the corresponding SOFA domain. The neurological domain was excluded because Glasgow Coma Scale assessment may be unreliable in sedated patients.

Abbreviations: FiO_2_, fraction of inspired oxygen; OF, organ failure; PaO_2_, partial pressure of arterial oxygen; SOFA, Sequential Organ Failure Assessment.

**CONSORT 2025 checklist of information to include when reporting a randomised trial***

| **Section / Topic** | **No** | **CONSORT 2025 checklist item description** | **Reported on page no.** |
| --- | --- | --- | --- |
| **Title and abstract** | | |  |
| Title and structured abstract | 1a | Identification as a randomised trial | 1, 2 |
|  | 1b | Structured summary of the trial design, methods, results, and conclusions | 2, 3 |
| **Open science** | | |  |
| Trial registration | 2 | Name of trial registry, identifying number (with URL) and date of registration | 2, 3 |
| Protocol and statistical analysis plan | 3 | Where the trial protocol and statistical analysis plan can be accessed | 6 |
| Data sharing | 4 | Where and how the individual de-identified participant data (including data dictionary), statistical code and any other materials can be accessed | 25 |
| Funding and conflicts of interest | 5a | Sources of funding and other support (e.g., supply of drugs), and role of funders in the design, conduct, analysis and reporting of the trial | 25 |
|  | 5b | Financial and other conflicts of interest of the manuscript authors | 25 |
| **Introduction** | | |  |
| Background and rationale | 6 | Scientific background and rationale | 4, 5 |
| Objectives | 7 | Specific objectives related to benefits and harms | 5 |
| **Methods** | | |  |
| Patient and public involvement | 8 | Details of patient or public involvement in the design, conduct and reporting of the trial | 6 |
| Trial design | 9 | Description of trial design including type of trial (e.g., parallel group, crossover), allocation ratio, and framework (e.g., superiority, equivalence, non-inferiority, exploratory) | 5, 6 |
| Changes to trial protocol | 10 | Important changes to the trial after it commenced including any outcomes or analyses that were not prespecified, with reason | 6 |
| Trial setting | 11 | Settings (e.g., community, hospital) and locations (e.g., countries, sites) where the trial was conducted | 5, 6 |
| Eligibility criteria | 12a | Eligibility criteria for participants | 6 |
|  | 12b | If applicable, eligibility criteria for sites and for individuals delivering the interventions (e.g., surgeons, physiotherapists) | 6 |
| Intervention and comparator | 13 | Intervention and comparator with sufficient details to allow replication. If relevant, where additional materials describing the intervention and comparator (e.g., intervention manual) can be accessed | 6 |
| Outcomes | 14 | Pre-specified primary and secondary outcomes, including the specific measurement variable (e.g., systolic blood pressure), analysis metric (e.g., change from baseline, final value, time to event), method of aggregation (e.g., median, proportion), and time point for each outcome | 7 |
| Harms | 15 | How harms were defined and assessed (e.g., systematically, non-systematically) | 7 |
| Sample size | 16a | How sample size was determined, including all assumptions supporting the sample size calculation | 10 |
|  | 16b | Explanation of any interim analyses and stopping guidelines | 6 |
| Randomisation: |  |  |  |
| Sequence generation | 17a | Who generated the random allocation sequence and the method used | 6 |
|  | 17b | Type of randomisation and details of any restriction (e.g., stratification, blocking and block size) | 6 |
| Allocation concealment mechanism | 18 | Mechanism used to implement the random allocation sequence (e.g., central computer/telephone; sequentially numbered, opaque, sealed containers), describing any steps to conceal the sequence until interventions were assigned | 6 |
| Implementation | 19 | Whether the personnel who enrolled and those who assigned participants to the interventions had access to the random allocation sequence | 6 |
| Blinding | 20a | Who was blinded after assignment to interventions (e.g., participants, care providers, outcome assessors, data analysts) | N/A |
|  | 20b | If blinded, how blinding was achieved and description of the similarity of interventions | N/A |
| Statistical methods | 21a | Statistical methods used to compare groups for primary and secondary outcomes, including harms | 8, 9, 10 |
|  | 21b | Definition of who is included in each analysis (e.g., all randomised participants), and in which group | 8, 9, 10 |
|  | 21c | How missing data were handled in the analysis | 8 |
|  | 21d | Methods for any additional analyses (e.g., subgroup and sensitivity analyses), distinguishing prespecified from post-hoc | 9-13 |
| **Results** | | |  |
| Participant flow, including flow diagram | 22a | For each group, the numbers of participants who were randomly assigned, received intended intervention, and were analysed for the primary outcome | 7, 13 |
|  | 22b | For each group, losses and exclusions after randomisation, together with reasons | 7 |
| Recruitment | 23a | Dates defining the periods of recruitment and follow-up for outcomes of benefits and harms | 7 |
|  | 23b | If relevant, why the trial ended or was stopped | N/A |
| Intervention and comparator delivery | 24a | Intervention and comparator as they were actually administered (e.g., where appropriate, who delivered the intervention/comparator, how participants adhered, whether they were delivered as intended [fidelity]) | 14, 15 |
|  | 24b | Concomitant care received during the trial for each group | 14, 15 |
| Baseline data | 25 | A table showing baseline demographic and clinical characteristics for each group | 13 |
| Numbers analysed,  outcomes and estimation | 26 | For each primary and secondary outcome, by group:   - the number of participants included in the analysis - the number of participants with available data at the outcome time point - result for each group, and the estimated effect size and its precision (such as 95% confidence interval) - for binary outcomes, presentation of both absolute and relative effect size | 14, 15 |
| Harms | 27 | All harms or unintended events in each group | 14 |
| Ancillary analyses | 28 | Any other analyses performed, including subgroup and sensitivity analyses, distinguishing pre-specified from post-hoc | 15-18 |
| **Discussion** | | |  |
| Interpretation | 29 | Interpretation consistent with results, balancing benefits and harms, and considering other relevant evidence | 18-22 |
| Limitations | 30 | Trial limitations, addressing sources of potential bias, imprecision, generalisability, and, if relevant, multiplicity of analyses | 22, 23, 24 |

*We strongly recommend reading this statement in conjunction with the CONSORT 2025 Explanation and Elaboration and/or the CONSORT 2025 Expanded Checklist for important clarifications on all the items. We also recommend reading relevant CONSORT extensions. See [www.consort-spirit.org](http://www.consort-spirit.org).

Citation: Hopewell S, Chan AW, Collins GS, Hróbjartsson A, Moher D, Schulz KF, et al. CONSORT 2025 Statement: updated guideline for reporting randomised trials. BMJ. 2025; 388:e081123. <https://dx.doi.org/10.1136/bmj-2024-081123>.

© 2025 Hopewell et al. This is an Open Access article distributed under the terms of the Creative Commons Attribution License (<https://creativecommons.org/licenses/by/4.0/>), which permits unrestricted use, distribution, and reproduction in any medium, provided the original work is properly cited.

**Supplementary Table S1. Nutrition Therapy Delivery and Target Achievement by Age Group and Treatment Allocation.**

|  | Age: ≤65 years (N = 1538) | | | Age: 66-75 years (N = 528) | | | Age: ≥76 years (N = 606) | | |
| --- | --- | --- | --- | --- | --- | --- | --- | --- | --- |
|  | Intervention  (N = 754) | Control  (N = 784) | P | Intervention  (N = 262) | Control  (N = 266) | P | Intervention  (N = 310) | Control  (N = 296) | P |
| **Nutrition therapy initiation and delivery** | | | | | | | | | |
| Time to initiation of nutrition therapy, day | 2.0 (1.0, 2.0) | 1.0 (1.0, 3.0) | 0.193 | 2.0 (1.0, 2.0) | 1.0 (1.0, 2.0) | 0.016 | 1.0 (1.0, 2.0) | 1.0 (1.0, 2.0) | < 0.001 |
| No EN / PN within 7 days, n (%) | 21 (2.8) | 30 (3.8) | 0.318 | 8 (3.1) | 9 (3.4) | 1.000 | 11 (3.5) | 16 (5.4) | 0.363 |
| Time to initiation of EN, day | 2.0 (1.0, 3.0) | 2.0 (1.0, 4.0) | < 0.001 | 2.0 (1.0, 3.0) | 2.0 (1.0, 3.0) | 0.607 | 2.0 (1.0, 2.0) | 2.0 (1.0, 3.0) | 0.115 |
| Time to initiation of PN, day | 1.0 (1.0, 3.0) | 1.0 (1.0, 2.0) | 0.005 | 2.0 (1.0, 3.0) | 1.0 (1.0, 2.0) | 0.006 | 2.0 (1.0, 3.0) | 1.0 (1.0, 1.0) | < 0.001 |
| Initiation of EN within 48 h (%) | 469 (62.2) | 383 (48.9) | < 0.001 | 177 (67.6) | 152 (57.1) | 0.017 | 228 (73.5) | 189 (63.9) | 0.013 |
| Initiation of PN within 48 h (%) | 165 (21.9) | 328 (41.8) | < 0.001 | 44 (16.8) | 100 (37.6) | < 0.001 | 40 (12.9) | 133 (44.9) | < 0.001 |
| Mean daily energy delivery over 7 days, kcal/kg/day | 14.4 (9.1, 20.0) | 13.2 (8.4, 19.4) | 0.040 | 14.3 (9.6, 19.1) | 14.7 (10.0, 20.6) | 0.329 | 15.2 (10.7, 19.9) | 15.8 (11.0, 20.7) | 0.245 |
| Mean daily protein delivery over 7 days, g/kg/day | 0.6 (0.4, 0.8) | 0.5 (0.3, 0.8) | 0.202 | 0.6 (0.4, 0.7) | 0.6 (0.4, 0.8) | 0.064 | 0.6 (0.4, 0.8) | 0.6 (0.4, 0.9) | 0.040 |
|  |  |  |  |  |  |  |  |  |  |
| **Achievement of EN targets** | | | | | | | | | |
| Day 7 EN energy target ratio, % | 72.7 (38.2, 100.1) | 57.1 (3.5, 85.7) | < 0.001 | 70.0 (33.3, 100.0) | 65.5 (26.8, 92.3) | 0.069 | 75.7 (50.0, 100.0) | 66.3 (28.6, 88.3) | < 0.001 |
| Day 7 EN protein target ratio, % | 55.4 (24.7, 76.9) | 40.0 (0.0, 66.7) | < 0.001 | 52.4 (23.5, 82.4) | 43.1 (14.6, 74.0) | 0.148 | 51.3 (33.3, 77.8) | 49.1 (20.8, 68.1) | 0.019 |
| Day 7 EN energy delivery ≥60% of target, n (%) | 474 (62.9%) | 372 (47.4%) | < 0.001 | 158 (60.3%) | 141 (53.0%) | 0.091 | 210 (67.7%) | 164 (55.4%) | 0.002 |
| Day 7 EN protein delivery ≥60% of target, n (%) | 340 (45.1%) | 245 (31.2%) | < 0.001 | 112 (42.7%) | 98 (36.8%) | 0.166 | 126 (40.6%) | 106 (35.8%) | 0.221 |
| Day 7 EN both energy and protein delivery ≥60% of target, n (%) | 334 (44.3%) | 229 (29.2%) | < 0.001 | 112 (42.7%) | 92 (34.6%) | 0.054 | 126 (40.6%) | 103 (34.8%) | 0.138 |

Continuous variables were compared using the Wilcoxon rank-sum test and are presented as median (interquartile range), while categorical variables were compared using the chi-square test or Fisher’s exact test, as appropriate, and are presented as number (percentage).

Abbreviations: EN, enteral nutrition; PN, parenteral nutrition.

**Supplementary Table S2. Proportion of missing values for baseline characteristics.**

| Variables | N (%) |
| --- | --- |
| APACHE II score | 40 (1.5) |
| mNUTRIC score | 42 (1.6) |
| Acute gastrointestinal injury grade | 25 (0.9) |

Abbreviations: APACHE II, Acute Physiology and Chronic Health Evaluation II; mNUTRIC, modified Nutrition Risk in Critically Ill.

**Supplementary Table S3. Proportional hazards assumption diagnostics for Cox proportional hazards models of 28-day all-cause mortality after ICU admission in different age groups.**

| Group | Variable | P Value |
| --- | --- | --- |
| ≤ 65 years | Intervention | 0.30 |
|  | Age | 0.06 |
|  | Sex | 0.55 |
|  | SOFA | 0.58 |
|  | BMI | 0.88 |
|  | GLOBAL | 0.25 |
|  |  |  |
| 66–75 years | Intervention | 0.10 |
|  | Age | 0.24 |
|  | Sex | 0.71 |
|  | SOFA | 0.93 |
|  | BMI | 0.07 |
|  | GLOBAL | 0.11 |
|  |  |  |
| ≥ 76 years | Intervention | 0.68 |
|  | Age | 0.75 |
|  | Sex | 0.19 |
|  | SOFA | 0.33 |
|  | BMI | 0.80 |
|  | GLOBAL | 0.73 |

Abbreviations: BMI, body mass index; SOFA, Sequential Organ Failure Assessment.

**Supplementary Table S4. Baseline characteristics of patients included in the present analysis and those excluded because of incomplete 28-day follow-up.**

| Characteristics | Included  (N = 2672) | Excluded  (N = 100) | SMD |
| --- | --- | --- | --- |
| Age, years | 62.0 (49.0, 74.0) | 64.0 (51.0, 73.0) | 0.026 |
| Male, n (%) | 1798 (67.3) | 68 (68.0) | 0.015 |
| Body mass index, kg/m^2^ | 22.9 (20.8, 24.6) | 22.0 (20.8, 23.7) | 0.161 |
| SOFA score | 7.0 (5.0, 10.0) | 3.0 (2.0, 6.0) | 0.874 |
| APACHE II score | 18.0 (14.0, 23.0) | 16.0 (13.0, 25.0) | 0.121 |
| mNUTRIC score | 4.0 (3.0, 6.0) | 5.0 (4.0, 7.0) | 0.409 |
| AGI grade, n (%) |  |  | 0.436 |
| AGI I | 1884 (71.2) | 23 (76.7) |  |
| AGI II | 523 (19.8) | 3 (10.0) |  |
| AGI III | 175 (6.6) | 1 (3.3) |  |
| AGI IV | 65 (2.5) | 3 (10.0) |  |
| Primary admission diagnosis, n (%) |  |  | 0.996 |
| Respiratory system | 824 (30.8) | 12 (12.0) |  |
| Cardiovascular system | 679 (25.4) | 20 (20.0) |  |
| Neurological system | 771 (28.9) | 12 (12.0) |  |
| Others | 398 (14.9) | 56 (56.0) |  |
| Source of ICU admission, n (%) |  |  | 0.778 |
| Emergency department | 1100 (41.2) | 27 (27.0) |  |
| Surgical department | 679 (25.4) | 18 (18.0) |  |
| Medical department | 462 (17.3) | 6 (6.0) |  |
| Others | 431 (16.1) | 49 (49.0) |  |
| Comorbidities, n (%) |  |  |  |
| Diabetes mellitus | 490 (18.3) | 11 (11.0) | 0.209 |
| Hypertension | 1172 (43.9) | 19 (19.0) | 0.556 |
| Coronary artery disease | 454 (17.0) | 10 (10.0) | 0.206 |
| Chronic respiratory disease | 262 (9.8) | 6 (6.0) | 0.141 |
| Stroke | 375 (14.0) | 11 (11.0) | 0.092 |
| Gastrointestinal disease | 196 (7.3) | 5 (5.0) | 0.097 |
| Malignant disease | 99 (3.7) | 3 (3.0) | 0.039 |
| Organ support therapy, n (%) |  |  |  |
| Continuous renal replacement therapy | 328 (12.3) | 3 (3.0) | 0.355 |
| Mechanical ventilation | 1866 (69.8) | 21 (21.0) | 1.125 |
| Vasopressors | 908 (34.0) | 16 (16.0) | 0.425 |

Values are presented as median (interquartile range) for continuous variables and number (percentage) for categorical variables.

Abbreviations: AGI, acute gastrointestinal injury; APACHE II, Acute Physiology and Chronic Health Evaluation II; ICU, intensive care unit; SOFA, Sequential Organ Failure Assessment.

**Supplementary Table S5. Daily gastrointestinal intolerance and feeding interruption during the first 7 days after ICU admission.**

|  | Age: ≤65 years (N = 1538) | | | Age: 66-75 years (N = 528) | | | Age: ≥76 years (N = 606) | | |
| --- | --- | --- | --- | --- | --- | --- | --- | --- | --- |
|  | Intervention  (N = 754) | Control  (N = 784) | P | Intervention  (N = 262) | Control  (N = 266) | P | Intervention  (N = 310) | Control  (N = 296) | P |
| Abdominal distension/pain |  |  |  |  |  |  |  |  |  |
| Day 1 | 28 (3.7) | 43 (5.5) | 0.125 | 15 (5.7) | 16 (6.0) | 1.000 | 18 (5.8) | 17 (5.7) | 1.000 |
| Day 2 | 70 (9.3) | 82 (10.5) | 0.492 | 24 (9.2) | 25 (9.4) | 1.000 | 25 (8.1) | 34 (11.5) | 0.199 |
| Day 3 | 102 (13.5) | 119 (15.2) | 0.395 | 35 (13.4) | 45 (16.9) | 0.308 | 25 (8.1) | 36 (12.2) | 0.123 |
| Day 4 | 90 (11.9) | 138 (17.6) | 0.002 | 30 (11.5) | 41 (15.4) | 0.227 | 33 (10.6) | 43 (14.5) | 0.187 |
| Day 5 | 103 (13.7) | 154 (19.6) | 0.002 | 32 (12.2) | 49 (18.4) | 0.063 | 31 (10.0) | 42 (14.2) | 0.145 |
| Day 6 | 96 (12.7) | 129 (16.5) | 0.046 | 28 (10.7) | 41 (15.4) | 0.138 | 35 (11.3) | 40 (13.5) | 0.479 |
| Day 7 | 92 (12.2) | 125 (15.9) | 0.042 | 27 (10.3) | 41 (15.4) | 0.105 | 38 (12.3) | 34 (11.5) | 0.867 |
| Any episode during Days 1–7 | 227 (30.1) | 267 (34.1) | 0.109 | 76 (29.0) | 86 (32.3) | 0.463 | 70 (22.6) | 75 (25.3) | 0.484 |
| ≥2 consecutive days with episode during Days 1–7 | 136 (18.0) | 173 (22.1) | 0.056 | 44 (16.8) | 49 (18.4) | 0.707 | 39 (12.6) | 52 (17.6) | 0.109 |
|  |  |  |  |  |  |  |  |  |  |
| Nausea/vomiting |  |  |  |  |  |  |  |  |  |
| Day 1 | 8 (1.1) | 3 (0.4) | 0.202 | 5 (1.9) | 0 (0.0) | 0.070 | 5 (1.6) | 0 (0.0) | 0.081 |
| Day 2 | 8 (1.1) | 6 (0.8) | 0.732 | 5 (1.9) | 2 (0.8) | 0.435 | 3 (1.0) | 2 (0.7) | 1.000 |
| Day 3 | 12 (1.6) | 12 (1.5) | 1.000 | 1 (0.4) | 5 (1.9) | 0.225 | 4 (1.3) | 6 (2.0) | 0.695 |
| Day 4 | 12 (1.6) | 9 (1.1) | 0.596 | 1 (0.4) | 3 (1.1) | 0.626 | 0 (0.0) | 6 (2.0) | 0.035 |
| Day 5 | 8 (1.1) | 10 (1.3) | 0.878 | 1 (0.4) | 6 (2.3) | 0.133 | 1 (0.3) | 5 (1.7) | 0.198 |
| Day 6 | 8 (1.1) | 9 (1.1) | 1.000 | 2 (0.8) | 3 (1.1) | 1.000 | 4 (1.3) | 3 (1.0) | 1.000 |
| Day 7 | 8 (1.1) | 10 (1.3) | 0.878 | 1 (0.4) | 1 (0.4) | 1.000 | 2 (0.6) | 4 (1.4) | 0.640 |
| Any episode during Days 1–7 | 47 (6.2) | 41 (5.2) | 0.461 | 14 (5.3) | 13 (4.9) | 0.968 | 16 (5.2) | 13 (4.4) | 0.800 |
| ≥2 consecutive days with episode during Days 1–7 | 10 (1.3) | 9 (1.1) | 0.932 | 1 (0.4) | 4 (1.5) | 0.378 | 1 (0.3) | 5 (1.7) | 0.198 |
|  |  |  |  |  |  |  |  |  |  |
| Diarrhoea |  |  |  |  |  |  |  |  |  |
| Day 1 | 14 (1.9) | 20 (2.6) | 0.452 | 12 (4.6) | 8 (3.0) | 0.472 | 13 (4.2) | 16 (5.4) | 0.611 |
| Day 2 | 69 (9.2) | 64 (8.2) | 0.550 | 24 (9.2) | 25 (9.4) | 1.000 | 37 (11.9) | 29 (9.8) | 0.475 |
| Day 3 | 92 (12.2) | 101 (12.9) | 0.744 | 38 (14.5) | 40 (15.0) | 0.960 | 48 (15.5) | 46 (15.5) | 1.000 |
| Day 4 | 140 (18.6) | 138 (17.6) | 0.670 | 52 (19.8) | 38 (14.3) | 0.113 | 74 (23.9) | 52 (17.6) | 0.070 |
| Day 5 | 171 (22.7) | 160 (20.4) | 0.307 | 49 (18.7) | 59 (22.2) | 0.377 | 86 (27.7) | 61 (20.6) | 0.051 |
| Day 6 | 163 (21.6) | 179 (22.8) | 0.609 | 61 (23.3) | 47 (17.7) | 0.136 | 77 (24.8) | 64 (21.6) | 0.401 |
| Day 7 | 170 (22.5) | 177 (22.6) | 1.000 | 62 (23.7) | 56 (21.1) | 0.538 | 76 (24.5) | 54 (18.2) | 0.075 |
| Any episode during Days 1–7 | 387 (51.3) | 386 (49.2) | 0.442 | 144 (55.0) | 137 (51.5) | 0.478 | 194 (62.6) | 149 (50.3) | 0.003 |
| ≥2 consecutive days with episode during Days 1–7 | 159 (21.1) | 163 (20.8) | 0.936 | 56 (21.4) | 47 (17.7) | 0.335 | 82 (26.5) | 60 (20.3) | 0.089 |
|  |  |  |  |  |  |  |  |  |  |
| Feeding interruption |  |  |  |  |  |  |  |  |  |
| Day 1 | 23 (3.1) | 14 (1.8) | 0.147 | 10 (3.8) | 6 (2.3) | 0.428 | 11 (3.5) | 3 (1.0) | 0.071 |
| Day 2 | 35 (4.6) | 20 (2.6) | 0.038 | 14 (5.3) | 7 (2.6) | 0.170 | 14 (4.5) | 2 (0.7) | 0.007 |
| Day 3 | 29 (3.8) | 32 (4.1) | 0.916 | 12 (4.6) | 15 (5.6) | 0.723 | 12 (3.9) | 5 (1.7) | 0.168 |
| Day 4 | 29 (3.8) | 44 (5.6) | 0.131 | 18 (6.9) | 16 (6.0) | 0.824 | 10 (3.2) | 5 (1.7) | 0.339 |
| Day 5 | 30 (4.0) | 48 (6.1) | 0.072 | 13 (5.0) | 18 (6.8) | 0.486 | 8 (2.6) | 5 (1.7) | 0.634 |
| Day 6 | 29 (3.8) | 49 (6.2) | 0.042 | 12 (4.6) | 10 (3.8) | 0.799 | 11 (3.5) | 5 (1.7) | 0.241 |
| Day 7 | 32 (4.2) | 30 (3.8) | 0.775 | 14 (5.3) | 14 (5.3) | 1.000 | 10 (3.2) | 4 (1.4) | 0.206 |
| Any episode during Days 1–7 | 128 (17.0) | 127 (16.2) | 0.733 | 51 (19.5) | 44 (16.5) | 0.446 | 43 (13.9) | 20 (6.8) | 0.006 |
| ≥2 consecutive days with episode during Days 1–7 | 27 (3.6) | 35 (4.5) | 0.453 | 14 (5.3) | 12 (4.5) | 0.810 | 15 (4.8) | 4 (1.4) | 0.026 |

**Supplementary Table S6. Nutrition related adverse events by age group and treatment allocation.**

|  | Age: ≤65 years (N = 1538) | | Age: 66-75 years (N = 528) | | Age: ≥76 years (N = 606) | |
| --- | --- | --- | --- | --- | --- | --- |
| Adverse events, n (%) | Intervention  (N = 754) | Control  (N = 784) | Intervention  (N = 262) | Control  (N = 266) | Intervention  (N = 310) | Control  (N = 296) |
| Gastrointestinal intolerance symptoms | 0 (0.0) | 0 (0.0) | 1 (0.4) | 0 (0.0) | 3 (1.0) | 0 (0.0) |
| Gastrointestinal bleeding | 0 (0.0) | 2 (0.3) | 0 (0.0) | 0 (0.0) | 0 (0.0) | 0 (0.0) |
| Aspiration events | 0 (0.0) | 1 (0.1) | 0 (0.0) | 1 (0.4) | 0 (0.0) | 0 (0.0) |
| Others | 1 (0.1) | 1 (0.1) | 0 (0.0) | 1 (0.4) | 0 (0.0) | 0 (0.0) |

**Supplementary Table S7. Knot selection for RCS models of age for 28-day all-cause mortality after ICU admission.**

| Number of knot | AIC | BIC | Non-linear P |
| --- | --- | --- | --- |
| 3 | 6047.73 | 6094.86 | 0.984 |
| 4 | 6049.00 | 6107.91 | 0.323 |
| 5 | 6048.79 | 6119.47 | 0.282 |
| 6 | 6051.36 | 6133.83 | 0.371 |

RCS models were fitted for age in multivariable mixed-effects Cox proportional hazards models for 28-day all-cause mortality after ICU admission, with adjustment for sex, body mass index, and Sequential Organ Failure Assessment Score.

Knots were placed at prespecified percentiles of the age distribution according to Harrell’s recommendations.

Model fit was assessed using the AIC and BIC, with lower values indicating better fit. Therefore, the three-knot RCS model was chosen.

Abbreviations: AIC, Akaike Information Criterion; BIC, Bayesian Information Criterion; ICU, intensive care unit; RCS, restricted cubic spline.

**Supplementary Table S8. 28-day all-cause mortality risks, weekly event rates, and rate differences after ICU admission by treatment group across age strata.**

| Age groups | Intervention | | | Control | | | Rate difference per week  (95% CI) (%)^a^ | P value^a^ |
| --- | --- | --- | --- | --- | --- | --- | --- | --- |
|  | No. of ICUs | No. of events, n (%) | Event rate  per week (%)^a^ | No. of ICUs | No. of events, n (%) | Event rate  per week (%)^a^ |  |  |
| ≤ 65 years | 43 | 64 (8.5) | 2.2 | 44 | 101 (12.9) | 3.5 | -1.23 (-2.10, -0.36) | 0.005 |
| 66-75 years | 40 | 42 (16.0) | 4.4 | 44 | 42 (15.8) | 4.3 | 0.13 (-1.73, 1.99) | 0.893 |
| ≥ 76 years | 42 | 82 (26.5) | 7.7 | 42 | 62 (20.9) | 5.8 | 1.83 (-0.38, 4.04) | 0.104 |

^a^Event rates were calculated as the number of deaths divided by total person-time at risk. Rate differences and their 95% confidence intervals were estimated assuming a Poisson distribution of events, with standard errors derived from event counts and person-time. Two-sided Z-tests were used to calculate P values.

The number of ICUs represents the number of clusters contributing patients to each age stratum within each treatment group. The intracluster correlation coefficients were 0.077 for 28-day all-cause mortality and 0.129 for age.

Abbreviations: CI, confidence interval; ICU, intensive care unit.

**Supplementary Table S9. Secondary outcomes by treatment group across age strata.**

| Secondary outcomes | Age group | Intervention | Control | P |
| --- | --- | --- | --- | --- |
| ICU length of stay, days | ≤ 65 years | 15.0 (8.0, 27.0) | 15.0 (9.0, 27.0) | 0.981 |
|  | 66-75 years | 13.0 (8.0, 25.0) | 14.0 (9.0, 28.0) | 0.039 |
|  | ≥ 76 years | 16.0 (10.0, 28.0) | 18.0 (10.0, 28.0) | 0.351 |
|  |  |  |  |  |
| ICU-free days within 28 days, days | ≤ 65 years | 10.0 (0.0, 19.0) | 10.0 (0.0, 19.0) | 0.174 |
|  | 66-75 years | 10.0 (0.0, 19.0) | 7.0 (0.0, 18.0) | 0.157 |
|  | ≥ 76 years | 0.0 (0.0, 15.0) | 0.0 (0.0, 16.0) | 0.822 |
|  |  |  |  |  |
| Persistent OF within 7 days | ≤ 65 years | 456 (60.5) | 542 (69.1) | < 0.001 |
|  | 66-75 years | 182 (69.5) | 196 (73.7) | 0.328 |
|  | ≥ 76 years | 201 (64.8) | 213 (72.0) | 0.072 |
|  |  |  |  |  |
| New-onset OF within 7 days | ≤ 65 years | 193 (25.6) | 215 (27.4) | 0.451 |
|  | 66-75 years | 71 (27.1) | 71 (26.7) | 0.994 |
|  | ≥ 76 years | 94 (30.3) | 98 (33.1) | 0.516 |
|  |  |  |  |  |
| Persistent multiple OF within 7 days | ≤ 65 years | 142 (18.8) | 231 (29.5) | < 0.001 |
|  | 66-75 years | 63 (24.0) | 65 (24.4) | 0.998 |
|  | ≥ 76 years | 56 (18.1) | 56 (18.9) | 0.868 |
|  |  |  |  |  |
| New-onset multiple OF within 7 days | ≤ 65 years | 36 (4.8) | 52 (6.6) | 0.145 |
|  | 66-75 years | 11 (4.2) | 12 (4.5) | 1.000 |
|  | ≥ 76 years | 23 (7.4) | 19 (6.4) | 0.745 |
|  |  |  |  |  |
| New-onset infection within 7 days | ≤ 65 years | 147 (19.5) | 183 (23.3) | 0.076 |
|  | 66-75 years | 41 (15.6) | 48 (18.0) | 0.536 |
|  | ≥ 76 years | 45 (14.5) | 42 (14.2) | 1.000 |

Continuous variables are presented as median (interquartile range), and categorical variables as number (percentage).

P values represent unadjusted between-group comparisons within each age stratum and were derived using tests appropriate to the distribution and type of each outcome. These descriptive comparisons differ from the adjusted mixed-effects regression analyses presented in Figure 4, which account for ICU-level clustering and adjust for age, sex, body mass index, and Sequential Organ Failure Assessment score. Two-sided P values are reported.

Abbreviations: ICU, intensive care unit; OF, organ failure.

**Supplementary Table S10. Per-Protocol analysis of the 28-day all-cause mortality after ICU admission using Cox proportional hazards models.**

| Age groups | aHR (95% CI)^a^ | P value^a^ | Interaction P |
| --- | --- | --- | --- |
| ≤ 65 years | 0.65 (0.44, 0.97) | 0.034 |  |
| 66-75 years | 0.93 (0.58, 1.48) | 0.751 | 0.041 |
| ≥ 76 years | 1.22 (0.82, 1.79) | 0.327 |  |

Per-protocol analysis was conducted in 2,531 patients.

^a^Estimates were derived from a mixed-effects Cox proportional hazards model including an interaction term between age group and treatment and adjusted for sex, body mass index, and Sequential Organ Failure Assessment score, with ICU included as a random effect to account for clustering.

Abbreviations: aHR, adjusted hazard ratio; CI, confidence interval; ICU, intensive care unit.

**Supplementary Table S11. Inverse-probability-of-censoring weighted sensitivity analysis for the per-protocol assessment of the age–treatment interaction on 28-day all-cause mortality after ICU admission.**

| Panel A: Continuous age–treatment interaction | aHR per 10-year increase in age (95% CI) |  | P for interaction |
| --- | --- | --- | --- |
| Age as a continuous variable | 1.26 (1.09, 1.46) |  | 0.001 |
|  |  |  |  |
| Panel B: Age-stratified treatment effects |  |  |  |
| Age group | aHR (95% CI) | P value | P for interaction |
| ≤65 years | 0.65 (0.44, 0.96) | 0.030 | 0.040 |
| 66–75 years | 0.85 (0.53, 1.38) | 0.512 |  |
| ≥76 years | 1.21 (0.83, 1.77) | 0.326 |  |

The per-protocol population excluded patients who died or were discharged from the ICU within 7 days after ICU admission. Inverse-probability-of-censoring weights were estimated using a logistic regression model for the probability of remaining eligible for the per-protocol analysis, including treatment allocation, age, sex, body mass index, Sequential Organ Failure Assessment score, mechanical ventilation, vasopressor use, and continuous renal replacement therapy. Weighted mixed-effects Cox proportional hazards models were fitted with ICU included as a random effect to account for within-ICU clustering. P values for treatment effects were derived from Wald tests, and P for interaction was obtained from joint Wald tests of the treatment-by-age interaction terms.

Abbreviations: aHR, adjusted hazard ratio; CI, confidence interval; ICU, intensive care unit.

**Supplementary Table S12. Sensitivity analysis of the 28-day all-cause mortality after ICU admission with incremental multivariable adjustment.**

| Models | Age groups | aHR (95% CI)^a^ | P value^a^ | Interaction P |
| --- | --- | --- | --- | --- |
| Base model^b^ + SOFA, primary admission diagnosis, AGI grade | ≤ 65 years | 0.64 (0.44, 0.93) | 0.020 |  |
|  | 66-75 years | 0.97 (0.62, 1.53) | 0.899 | 0.013 |
|  | ≥ 76 years | 1.33 (0.93, 1.91) | 0.114 |  |
|  |  |  |  |  |
| Base model^b^ + APACHE II, mNUTRIC | ≤ 65 years | 0.65 (0.44, 0.94) | 0.021 |  |
|  | 66-75 years | 0.91 (0.56, 1.49) | 0.719 | 0.026 |
|  | ≥ 76 years | 1.24 (0.84, 1.85) | 0.282 |  |
|  |  |  |  |  |
| Base model^b^ + SOFA, CRRT, MV, vasopressors | ≤ 65 years | 0.68 (0.47, 0.99) | 0.043 |  |
|  | 66-75 years | 1.04 (0.65, 1.65) | 0.883 | 0.011 |
|  | ≥ 76 years | 1.34 (0.92, 1.94) | 0.127 |  |
|  |  |  |  |  |
| Base model^b^ + primary admission diagnosis, AGI grade, APACHE II, mNUTRIC, CRRT, MV, vasopressors | ≤ 65 years | 0.69 (0.50, 0.98) | 0.047 |  |
|  | 66-75 years | 0.95 (0.59, 1.53) | 0.819 | 0.029 |
|  | ≥ 76 years | 1.33 (0.92, 1.92) | 0.134 |  |

^a^Estimates were derived from mixed-effects Cox proportional hazards models including an interaction term between age group and treatment, with ICU included as a random effect.

^b^Base model adjusted for age, sex, and body mass index.

For models containing APACHE II and mNUTRIC, SOFA score was omitted to avoid simultaneous adjustment for overlapping measures of illness severity.

Abbreviations: aHR, adjusted hazard ratio; APACHE II, Acute Physiology and Chronic Health Evaluation II; CI, confidence interval; CRRT, continuous renal replacement therapy; ICU, intensive care unit; mNUTRIC, modified Nutrition Risk in Critically Ill; SOFA, Sequential Organ Failure Assessment.

**Supplementary Table S13. Distribution of patients lost to follow-up and extreme-case sensitivity analyses for 28-day all-cause mortality after ICU admission.**

| Panel A. Patients lost to follow-up according to age group and treatment assignment | | | | |
| --- | --- | --- | --- | --- |
| Age group | Intervention | Control | Total | |
| ≤ 65 years | 41 | 16 | 57 | |
| 66-75 years | 17 | 7 | 24 | |
| ≥ 76 years | 15 | 4 | 19 | |
|  | 73 | 27 | 100 | |
| Panel B. Extreme-case sensitivity analyses for 28-day mortality among patients aged ≤ 65 years | | | | |
| Scenario | Intervention | Control | Risk difference, percentage points | Risk ratio |
| Observed analysis | 64/754 (8.5%) | 101/784 (12.9%) | -4.39 | 0.66 |
| All lost assumed to have survived | 64/795 (8.1%) | 101/800 (12.6%) | -4.57 | 0.64 |
| All lost assumed to have died | 105/795 (13.2%) | 117/800 (14.6%) | -1.42 | 0.90 |
| Best case for intervention^*^ | 64/795 (8.1%) | 117/800 (14.6%) | -6.57 | 0.55 |
| Worst case for intervention^†^ | 105/795 (13.2%) | 101/800 (12.6%) | 0.58 | 1.05 |

Values are n/N (%), unless otherwise indicated. Risk differences were calculated as the mortality risk in the intervention group minus that in the control group.

^*^Best-case scenario for the intervention assumes that all patients lost to follow-up in the intervention group survived and all those in the control group died.

^†^Worst-case scenario for the intervention assumes that all patients lost to follow-up in the intervention group died and all those in the control group survived.

**Supplementary Table S14. Sensitivity analyses accounting for randomisation stratification factors for 28-day all-cause mortality after ICU admission.**

| Models | Age specification | aHR (95% CI)^a^ | P value^a^ | Interaction P |
| --- | --- | --- | --- | --- |
| Base model^b^ + province and ICU type as fixed effects | Treatment × age,  per 10-year increase | 1.30 (1.15, 1.48) |  | < 0.001 |
|  | ≤ 65 years | 0.70 (0.49, 0.99) | 0.041 |  |
|  | 66-75 years | 0.90 (0.54, 1.49) | 0.672 | 0.004 |
|  | ≥ 76 years | 1.28 (0.86, 1.88) | 0.220 |  |
|  |  |  |  |  |
| Base model^b^ + province and ICU type as random effects | Treatment × age,  per 10-year increase | 1.29 (1.13, 1.46) |  | < 0.001 |
|  | ≤ 65 years | 0.64 (0.44, 0.91) | 0.015 |  |
|  | 66-75 years | 0.99 (0.62, 1.57) | 0.962 | 0.010 |
|  | ≥ 76 years | 1.29 (0.89, 1.87) | 0.182 |  |

ᵃEstimates were derived from mixed-effects Cox proportional hazards models. For the continuous-age analysis, the aHR represents the relative change in the treatment hazard ratio per 10-year increase in age, with the corresponding treatment-by-age interaction P value. For the age-group analysis, aHRs represent the intervention versus control treatment effect within each age group, and the interaction P value was derived from a joint Wald test of the treatment-by-age-group interaction terms. In the fixed-effects specification, province and ICU type were included as fixed effects, with ICU included as a random effect. In the random-effects specification, province, ICU type, and ICU were included as random effects.

ᵇBase model adjusted for age, sex, body mass index, and Sequential Organ Failure Assessment score.

Abbreviations: aHR, adjusted hazard ratio; CI, confidence interval; ICU, intensive care unit.

**Supplementary Figure S1. Implementation of the evidence-based feeding protocol.** Panel A: Algorithm of the evidence-based feeding protocol. Feeding intolerance was evaluated using the FIS. Panel B: Management of feeding intolerance. FIS is shown in Supplementary Methods. AGI, acute gastrointestinal injury; CD, Clostridium difficile; D/C, discontinue; EN, enteral nutrition; FIS, feeding intolerance score; GI, gastrointestinal; PN, parenteral nutrition; RBC, red blood cells; WBC, white blood cells.

**Supplementary Figure S2. Treatment effect on the primary outcome as a function of age as a continuous variable in the per-protocol analysis.** The figure shows the adjusted HR for the primary outcome (28-day all-cause mortality after ICU admission) associated with the intervention across age as a continuous variable in the per-protocol population. Estimates were derived from a mixed-effects Cox proportional hazards model including an interaction term between age and treatment, adjusted for sex, body mass index, and Sequential Organ Failure Assessment score, with ICU modelled as a random effect to account for clustering. The solid line represents the point estimate, and the shaded area indicates the 95% CI. CI, confidence interval; HR, hazard ratio.
